# Active lifestyle enhances protein expression profile in subjects with Lewy body pathology

**DOI:** 10.1590/1980-57642021dn15-010004

**Published:** 2021

**Authors:** Caroline Cristiano Real, Cláudia Kimie Suemoto, Karina Henrique Binda, Lea Tenenholz Grinberg, Carlos Augusto Pasqualucci, Wilson Jacob, Renata Eloah de Lucena Ferretti-Rebustini, Ricardo Nitrini, Renata Elaine Paraizo Leite, Luiz Roberto de Britto

**Affiliations:** 1 Laboratoy of Nuclear Medicine, Faculdade de Medicina, Universidade de São Paulo – São Paulo, SP, Brazil.; 2 Laboratory of Cellular Neurobiology, Department of Physiology and Biophysics, Instituto de Ciencias Biomedicas, Universidade de São Paulo – São Paulo, SP, Brazil.; 3 Division of Geriatrics, Faculdade de Medicina, Universidade de São Paulo – São Paulo, SP, Brazil.; 4 Memory and Aging Center, University of California San Francisco – San Francisco, California, United States of America.; 5 Department of Pathology, Faculdade de Medicina, Universidade de São Paulo – São Paulo, SP, Brazil.; 6 Medical-Surgical Nursing Department, Escola de Enfermagem, Universidade de São Paulo – São Paulo, SP, Brazil.; 7 Department of Neurology, Faculdade de Medicina, Universidade de São Paulo – São Paulo, SP, Brazil.

**Keywords:** Life style, aging, Lewy bodies, postmortem examination, Parkinson disease, dopamine, astrocytes, estilo de vida, envelhecimento, corpos de Lewy, autópsia, doença de Parkinson, dopamina, astrócitos

## Abstract

Clinical trials of the effects of physical activity have reported improvements in symptoms and quality of life in patients with Parkinson's disease (PD). Additionally, morphological brain changes after exercising were reported in PD animal models. However, these lifestyle-related changes were not evaluated in postmortem brain tissue.

**Objective:**

We aimed to evaluate, by immunohistochemistry, astrocytes, tyrosine hydroxylase (TH) and structural proteins expression (neurofilaments and microtubules — MAP2) changes in postmortem brain samples of individuals with Lewy body pathology.

**Methods:**

Braak PD stage≥III samples, classified by neuropathology analysis, from The Biobank for Aging Studies were classified into active (n=12) and non-active (n=12) groups, according to physical activity lifestyle, and paired by age, sex and Braak staging. Substantia nigra and basal ganglia were evaluated.

**Results:**

Groups were not different in terms of age or gender and had similar PD neuropathological burden (p=1.00). We observed higher TH expression in the active group in the substantia nigra and the basal ganglia (p=0.04). Astrocytes was greater in the non-active subjects in the midbrain (p=0.03) and basal ganglia (p=0.0004). MAP2 levels were higher for non-active participants in the basal ganglia (p=0.003) and similar between groups in the substantia nigra (p=0.46). Neurofilament levels for non-active participants were higher in the substantia nigra (p=0.006) but not in the basal ganglia (p=0.24).

**Conclusion:**

Active lifestyle seems to promote positive effects on brain by maintaining dopamine synthesis and structural protein expression in the nigrostriatal system and decrease astrogliosis in subjects with the same PD neuropathology burden.

## RESUMO.

Estudos dos efeitos da atividade física relataram melhora nos sintomas e na qualidade de vida de pacientes com doença de Parkinson (DP). Além disso, alterações morfológicas do cérebro após o exercício físico foram relatadas em modelos animais da DP. No entanto, essas mudanças relacionadas ao estilo de vida não foram avaliadas em tecido cerebral *post-mortem*.

**Objetivo:**

Avaliar a expressão de astrócitos, tirosina hidroxilase (TH) e a expressão de proteínas estruturais (neurofilamentos e microtúbulos — MAP2) por imuno-histoquímica, em amostras cerebrais *post-mortem* de indivíduos com corpos de Lewy.

**Métodos:**

Amostras com estágio de Braak para DP≥III, classificação neuropatológica, fornecidas pelo biobanco de estudos do envelhecimento foram classificadas em grupos ativos (n=12) e não ativos (n=12), de acordo com o estilo de vida (atividade física), e pareados por idade, sexo e estadiamento de Braak. Analisou-se a substância negra e gânglios da base.

**Resultados:**

Idade, sexo e classificação para DP foram semelhantes (p=1,00). Observou-se maior expressão de TH no grupo ativo (p=0,04). Amostras de não ativos revelaram maior expressão de astrócitos no mesencéfalo (p=0,03) e nos gânglios da base (p=0,0004); MAP2 nos gânglios da base (p=0,003); os níveis de neurofilamentos foram maiores na substância negra (p=0,006).

**Conclusão:**

O estilo de vida ativo parece promover efeitos positivos no cérebro, mantendo a síntese de dopamina e a expressão estrutural de proteínas no sistema nigrostriatal e com diminuição da ativação de astrócitos em indivíduos com a mesma classificação neuropatológica para a DP.

## INTRODUCTION

Although Parkinson disease (PD) is the most common form of α-synucleinophaties with movement disorder, and second most common neurodegenerative disorder worldwide, doubts about PD are still unclear.^1^ PD neuropathology classification is based on the detection of altered α-synuclein, responsible for the formation of Lewy bodies in the tissue and neuroanatomic distribution of this alteration in the brain, as proposed by the Braak staging criteria for PD,^2^ classifying it as a Lewy body pathology (LBP).^1^ Several studies have shown a variety of possible mechanisms for PD, including increased oxygen free radicals, mitochondrial dysfunction, protein degradation and aggregation dysfunction, and neuroinflammation,^3^ which is responsible for promoting the death of dopaminergic cells. The levels of tyrosine hydroxylase (TH) are severely reduced in the substantia nigra (SN) of PD patients.^4^ TH is decreased after dopaminergic cell death, thus it is considered a good index of dopaminergic function in postmortem studies.^4^

PD is increasingly thought to be associated with glial pathology. Astrocytes, the most present class of glial cells in the mammalian central nervous system (CNS), has been highlighted as key molecule of neuroinflammation and has a very heterogeneous functional level.^5^ Glial fibrillary acidic protein (GFAP) is a protein expressed in astrocytes widely described for having a relationship with neurodegenerative disease progression and CNS injuries,^6^ as well as dysregulation of nervous system homeostasis.^7^ Recently, research on neurodegenerative disorders has focused on understanding the role of astrogliosis in the disease pathophysiology, and has also been seem as a promising cellular source not only to study CNS pathologies initiation and progression, but also as a therapeutic target.^8^

In addition, structural proteins, such as microtubules and neurofilaments, are also important to be evaluated due to their importance for neuronal integrity and function. Previous studies revealed that, in neurons, microtubules maintain the integrity of axons by forming stable bundles and facilitate the transport of synaptic vesicles. One of the mechanisms used by cells to regulate the stability and dynamics of microtubules involves the interaction of microtubules with microtubule-associated proteins (MAP), including microtubule-associated protein-2 (MAP2). Interaction between MAP have either stabilizing or destabilizing effects on the microtubules.^9^ MAP2 is the major neuronal component, providing structural support for the axon and regulating its diameter, therefore, dysfunction in their synthesis can directly affect neurotransmission.^9^ Abnormal patterns of MAP2 in PD brain tissue have also been observed, and can be responsible for the destabilization of microtubule structures.^10^ In neurodegenerative diseases, including PD, microtubules destabilization may be a vital step in the pathogenesis.^11^

Neurofilaments, whose subunits have different domain structures and function, are the only neuron-specific intermediate filaments.^12^ They are important to give shape to cells; to determine axonal caliber, which controls signal conduction; to regulate the transport of synaptic vesicles; and to modulate synaptic plasticity.^13^ On the other hand, the accumulation of neurofilament proteins can develop aggregates, being one of the responsible for the formation of Lewy bodies in PD.^13^

Several studies have investigated interventions aimed to improve the quality of life of PD patients. Physical activity in older people is important to prevent the disease,^14^ as was seen in a recent longitudinal study with self-reported physical activity that revealed a decline in the clinical progression of PD.^15^ In PD animal models, we have previously shown that exercise protocols promoted a reduction in inflammatory markers,^16^ and an increase in dopamine function.^17^ Despite the symptomatic improvements found in clinical trials, studies of human postmortem brains correlating physical activity with morphological changes are lacking.

Therefore, we aimed to evaluate, in a human postmortem brain tissue with proven Lewy body pathology classified as PD by Braak criteria, the association between active or non-active lifestyle and structural proteins (MAP2 and neurofilaments) and astrogliosis, evaluated by GFAP. The dopaminergic system function was also investigated by measuring the TH protein expression in these individuals.

## METHODS

### Participants

From 2004 to 2016, the Brain Bank from the Biobank for Aging Studies (BB-BAS) included 1,123 participants. The inclusion criteria is to be 50 years old or older. Exclusion criteria included acute brain lesions (*e.g.*, infarctions, hemorrhages, cancer, or trauma); severe chronic conditions that could interfere in brain homeostasis (*e.g.*, severe heart failure and dialytic chronic kidney failure); subjects without a reliable next-of-kin to answer the clinical interview; and subjects with acidosis due to severe agonal status (cerebrospinal fluid pH<6.5).^18^ For this study, all participants with a neuropathological diagnosis of LBP classified as Braak PD stage≥III were included. Participants with incomplete information about lifestyle and bedridden were excluded. The deceased's next-of-kin signed an informed consent to donate the brain. All procedures were approved by local and national human research ethics committees (Certificate number 285/04, and 1181 CEPSH/ICB/USP).

### Postmortem clinical evaluation

Clinical assessment was obtained through a clinical interview with the next-of-kin. The protocol included semi-structured questionnaires that assessed clinical-functional and neuropsychiatric abilities, which were validated for postmortem interviews.^19^ The interview included the Tanner questionnaire, a brief, sensitive and specific screening questionnaire for parkinsonism, and the Clinical Dementia Rating (CDR) scale for cognitive evaluation. The Tanner questionnaire contains nine questions about parkinsonism symptoms (one point for each symptom). The CDR evaluates the presence and severity of cognitive impairment by assessing six domains: memory, orientation, judgment and problem solving, community affairs, home and hobbies, and personal care.

Because of the nature of this postmortem study, only the informant section of the CDR was used. Participants were then classified into five categories: normal cognition (CDR=0); questionable dementia (CDR=0.5); mild dementia (CDR=1); moderate dementia (CDR=2); and severe dementia (CDR=3). Subjects with CDR>0 were characterized with cognitive impairment. Participants were considered to have an active lifestyle if the next-of-kin reported that the subject had a walking routine of at least three times a week in the last 12 months before death, and was active in household activities. The non-active individuals were paired with active ones based on age, gender, and PD Braak staging.^18^ These information were obtained from the BB-BAS database.

### Neuropathological evaluations

Brain tissue was obtained within 24 hours after death. One hemisphere was fixed in 4% buffered paraformaldehyde and specific areas were embedded in paraffin: middle frontal gyrus, middle and superior temporal gyri, angular gyrus, superior frontal, anterior cingulate gyrus, visual cortex, hippocampal formation at the level of the lateral geniculate body, amygdala, basal ganglia at the level of the anterior commissure, thalamus, midbrain, pons, medulla oblongata, and cerebellum. Blocks were sectioned into 5-μm-thick sections stained with hematoxylin and eosin. Immunohistochemistry was performed with antibodies against β-amyloid (4G8, 1:10,000; Signet Pathology Systems, Dedham, Massachusetts), phosphorylated tau (PHF-1, 1:2,000; gift from Peter Davies, New York), TDP-43 (1:500, Proteintech, Chicago, Illinois), and α-synuclein (EQV-1, 1:10,000; gift from Kenji Ueda, Tokyo, Japan).^20^ Internationally accepted neuropathological criteria were used to stage and diagnose the brain pathologies.^21^ ^–^ ^25^ We considered the diagnosis of Lewy body disease or PD when Braak PD stage≥3.^25^

**Figure 1:**
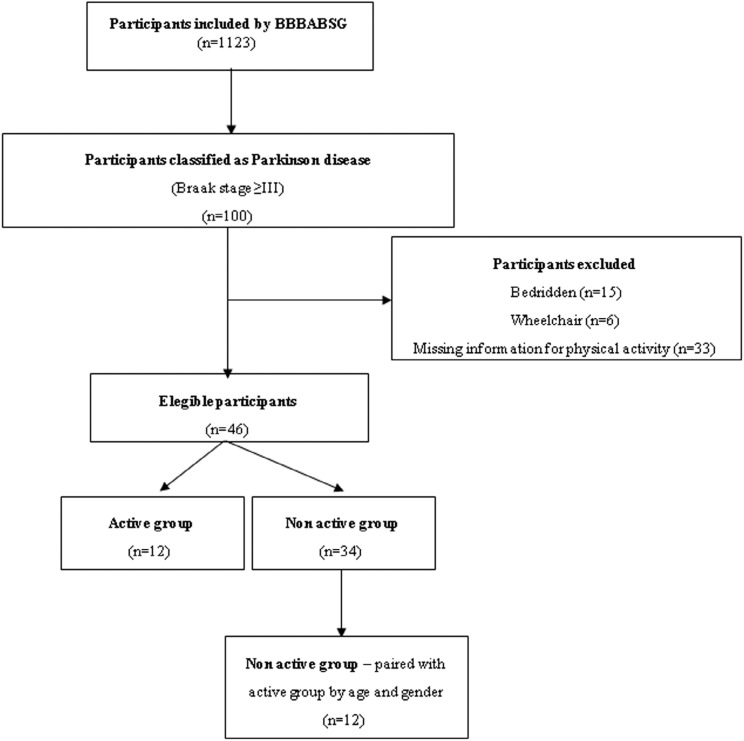
Flowchart of the inclusion process of the study participants.

The neuropathological classification was performed on all brains donated to BB-BAS. Based on the neuropathological classification, 100 participants were diagnosed with PD. Among these participants, 12 subjects were classified as active, and 34 were non-active. From the non-active group, 12 cases were selected and paired by age and gender with the active group. Therefore, this study included 24 subjects divided between active (n=12) and non-active groups (n=12) (Figure 1). The two groups had similar Braak PD staging (p=1.00). In addition, some participants also had Alzheimer disease (AD) neuropathology, but Braak staging for AD was similar between groups (p=1.00).

Selected samples were submitted to immunohistochemistry to evaluate structural proteins (neurofilaments and MAP2), astrogliosis (GFAP), and dopaminergic system (TH) in the midbrain (including the SN) and the basal ganglia (Caudate and Putamen nuclei). For that, slides with brain tissue were submitted to antigenic-retrieval treatment by immersion in citrate buffer pH 6.0 in a steamer at 95°C for 45 min.^26^ The sections were incubated overnight at 4°C with primary antibodies, namely: TH (Millipore, MAB5280, 1:2,000), neurofilaments (Zymed, 18-0171, 1:2,000); MAP2 (Millipore, MAB3418, 1:2,000) and GFAP (Sigma, G3893, 1:2,000). After secondary antibody incubation (Jackson ImmunoResearch Laboratories, 715-065-151, 1:200), the sections were incubated for 1 h at 37°C with avidin-biotin-peroxidase complex (Vectastain^®^ ABC Kit; Vector Laboratories, 1:100), and the slices were incubated with 0.05% 3,3’-diaminobenzidine tetrahydrochloride and a 0.01% solution of hydrogen peroxide in phosphate buffer. Intensification of the reaction was performed using 0.05% osmium tetroxide in water. The sections were dehydrated, cleared with xylol, and coverslipped with Permount (Fisher).

Five selected digital images with 10x magnification were acquired using a Nikon E1000 microscope (Melville, NY, USA) and a Nikon DMX1200 digital camera (Nikon Imaging Software ACT-U). An independent researcher blinded to the subject's physical activity status analyzed the integrated optical density of immunolabeling in five areas of 0.58 mm^2^ from each digital image, totaling an analysis area of 3 mm^2^ for each sample. Digital images were analyzed using the ImageJ software, version 1.52a (NIH, USA). For image analysis, the digital image was opened, a square of 0.58 mm^2^ was drawn, and the analysis instrument was used (Set Measurements – Integrated Density – Measure).

### Statistical analysis

Data are represented as mean and standard deviations for continuous variables and frequencies for categorical variables. As the active and non-active groups were paired, we used paired *t*-tests to compare protein expression between groups. The McNemar test was used to compare categorical variables. P<0.05 was adopted.

## RESULTS

Table 1 describes the characteristics of the samples. Tanner scores showed fewer parkinsonism symptoms among active participants compared to non-active ones (p=0.03). The non-active group revealed four subjects classified as CDR 0, three classified as CDR 0.5, one as CDR 1, two subjects as CDR 2, and two subjects as CDR 3. For the active group, there were seven subjects classified as CDR 0, one classified as CDR 1, one subject as CDR 2, and three as CDR 3 (p=0.45).

**Table 1:** Characteristics of the sample (n=24).

	Non-active (n=12)	Active (n=12)	p-value
Age (years), mean±SD*	80.8±2.42	81.2±2.52	0.71
Male, n (%)†	7 (58.3)	7 (58.3)	1.00
Braak PD staging≥III, n (%)†	12 (100)	12 (100)	1.00
Clinical Dementia Rating CDR>0, n (%)†	8 (66.7)	5 (41.7)	0.45
Braak AD staging≥III, n (%)†	9 (75)	9 (75)	1.00
Tanner score, mean±SD*	3.42±3.20	0.67±1.07	0.03

* paired t-test

† McNemar test; PD: Parkinson's disease; AD: Alzheimer's disease; SD: standard deviation.

**Figure 2:**
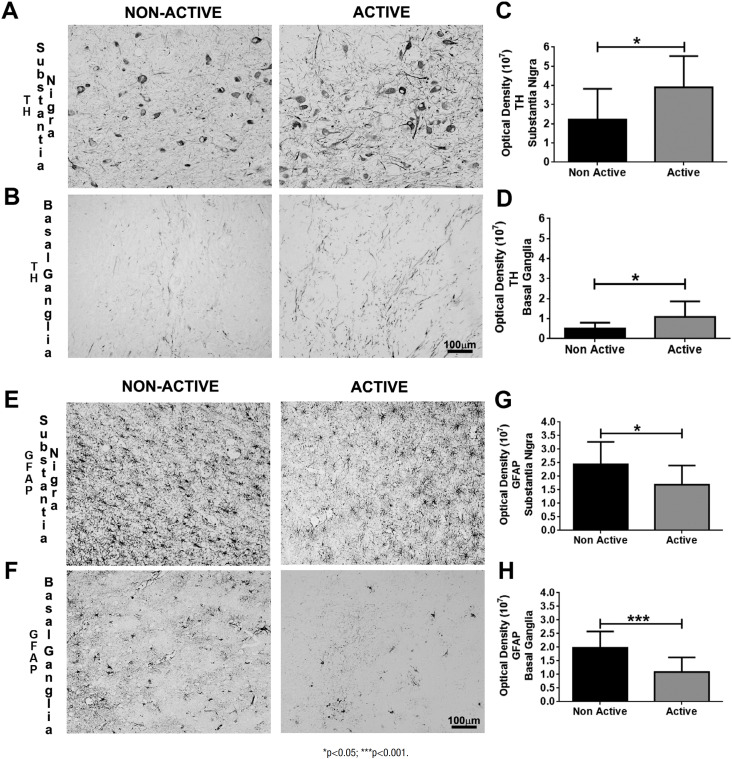
Digital images and graphics for tyrosine hydroxylase in the substantia nigra (A, C) and basal ganglia (B, D); and for glial fibrillary acidic protein in the substantia nigra (E and G) and basal ganglia (F and H).

Table 2 describes the mean and standard deviation of integrated density for each protein analyzed. TH was more highly expressed in the active group than in the non-active one in the SN (p=0.04) (Figures 2A and 2C) and in the basal ganglia (p=0.04) (Figures 2B and 2D). On the other hand, astrocyte was decreased in the substantia nigra of the active group compared to the non-active one (p=0.03) (Figures 2E and 2G) and increased in the basal ganglia among the non-active participants compared to the active participants (p<0.001) (Figures 2F and 2H).

**Table 2:** Protein expression in the substantia nigra and basal ganglia according to physical activity status (n=24).

	Substantia Nigra		Basal Ganglia	
	Non-active (n=12)	Active (n=12)	Non-active (n=12)	Active (n=12)
Tyrosine hydroxylase	2.21±1.62	3.90±1.63	0.51±0.28	1.09±0.77
mean±SD (10^7^)*				
Microtubule-associated protein 2	1.86±1.05	1.54±0.81	1.57±0.73	0.73±0.41
mean±SD (10^7^)*				
Neurofilaments	1.07±0.43	0.58±0.24	2.53±1.32	3.28±1.43
mean±SD (10^7^)*				
Glial fibrillary acidic protein	2.44±0.83	1.69±0.70	1.99±0.60	1.09±0.54
mean±SD (107)*				

* paired t-test. Data are expressed as the integrated density mean in an area of 0.58 mm^2^.

**Figure 3:**
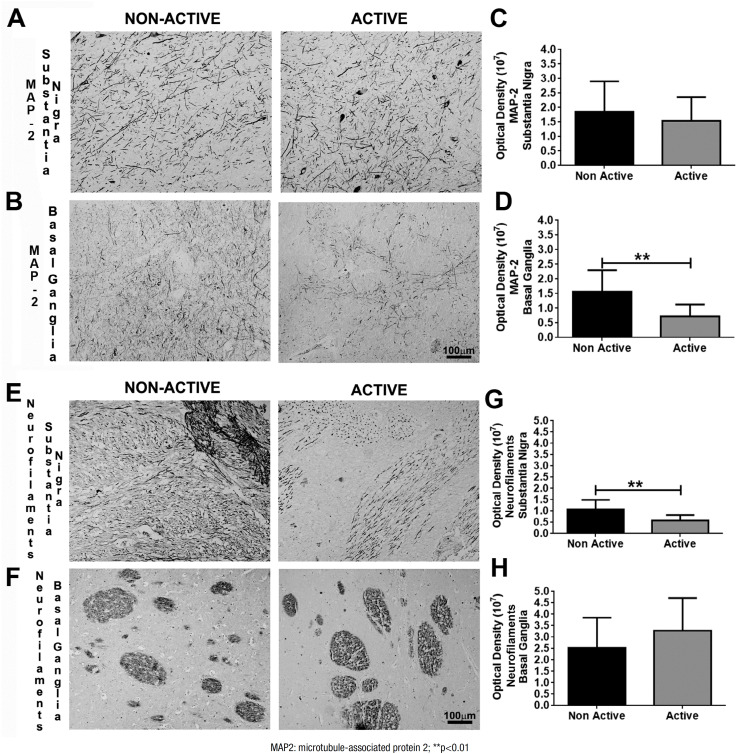
Digital images and graphics for microtubule-associated protein 2 in the substantia nigra (A, C) and basal ganglia (B, D); and for neurofilaments in the substantia nigra (E, G) and basal ganglia (F, H).

For MAP2, there were no differences between the groups in the substantia nigra (p=0.46) (Figures 3A and 3C), while the expression was higher in the non- active group than in the active group in the basal ganglia (p=0.003) (Figures 3B and 3D). Neurofilaments were more highly expressed in the non-active group than in the active one in the SN (p=0.006) (Figures 3E and 3G), while no differences were observed between groups in the basal ganglia (p=0.24) (Figures 3F and 3H).

## DISCUSSION

The present study is the first to correlate protein expression in individuals with proved LBP classified as PD, with an active lifestyle using human brain tissue. The active group, which included participants who walked at least three times a week during the year prior to death, showed higher expression of TH and lower expression of astrocytes in the midbrain and basal ganglia areas. On the other hand, MAP2 and neurofilament expression were higher in non-active subjects. Thus, this study can help explain the effects of physical activity on clinical progression improvement in PD patients.

It is important to reinforce that despite the small sample of subjects, all tissue samples analyzed in our study included only PD subjects with Braak stage≥III, and with the same Braak AD between groups confirmed by neuropathological classification. In addition, the Braak staging for PD was similar between groups, however the Tanner scale revealed more parkinsonism symptoms in the non-active participants, corroborating previous studies.^27^

Physical activity can slow down the aging process, which involves astrocytic,^28^ as well as promotes better motor learning capacity, through increased plasticity in motor-related structures.^29^ Progressive resistance exercise in PD patients with akinesia and rigidity can improve static posturography, gait, and quality of life.^30^ Comparisons between patients with PD who self-reported regular exercise (≥2.5 hours per week) and people who exercise <2.5 hours per week revealed positive effects of exercise on health-related quality of life and mobility changes over two years. The benefit of exercise on health-related quality of life was greater in advanced than mild PD.^31^ A recent clinical trial with 128 PD patients, followed for 3 years and with half the subjects undergoing exercise protocols, demonstrated that a high-intensity treadmill exercise protocol was able to promote improvement in Unified Parkinson's Disease Rating Scale motor scores.^32^

The current findings on TH expression in human postmortem material corroborate our previous studies with a 6-hydroxydopamine PD-like animal model. In animal models, treadmill exercise 3 times/week for 40 minutes led to the neuroprotection of the dopaminergic system with high TH expression in exercised animals, regardless of the exercise protocol.^16^ ^,^ ^17^ Despite the ability of different exercise protocols to protect the dopaminergic system and benefit the nervous system, better effects are found when the exercise begins earlier.^15^ In addition to high TH expression in the postmortem material from active participants, it was possible to observe that astrocytic activation was related to an increase in GFAP expression, which was also found in a previous study.^33^ Astrocyte dysfunction has been described in LBP given the critical role of this glial subtype in the metabolic and structural support of the nervous system.^7^

Studies suggest that astrocytes play an important role in the pathology and propagation of PD, since they sustain a hazardous environment and further promote dopaminergic neurodegeneration. The pathology of PD is not limited to neurons but extends to astrocytes, since the accumulation of α-synuclein is not only found in neurons, but also extends to astrocytes, which are capable of clearing α-synuclein deposits from neurons. After taking up α-synuclein, astrocytes are proposed to release cytokines including tumor necrosis factor and interleukin-6, thereby causing inflammatory responses, which can promote PD progression.^5^ However, the exact role of these cells on the pathophysiology of PD is still controversial, since they can also be active anti-inflammatory pathways.^8^ Nevertheless, the findings from PD postmortem studies on GFAP expression are inconsistent, since some have revealed GFAP upregulation and typical reactive morphology and others, minimal or mild astrogliosis in patients with PD.^5^ The finding of decreased GFAP expression in active participants corroborated with studies in PD-like rat models that have described reduced GFAP, dopaminergic neuron protection, and improvements in motor behavior after a treadmill exercise protocol.^16^

In human postmortem material, there is a decrease in axonal transport in subjects in early phases of PD when compared to subjects without dopaminergic degeneration. In contrast, in the symptomatic phase of PD, there is an increase in MAP2 and neurofilaments.^34^ These proteins, in the presence of α-synuclein protein, worsen clinical symptoms.^10^ In addition, the present study evidenced reduced expression of these proteins in the active group, which could be related to fewer symptoms of parkinsonism and better TH expression. AD-brain-isolated tau protein was also observed to co-stain with both endogenous tau and MAP2, suggesting sequestration of these proteins, and α-synuclein also binds MAP2 *in vitro*. The sequestration of other MAPs may play an important role in the ability of the hyperphosphorylated Tau protein to cause microtubules instability and neurodegeneration, suggesting that the non-active group may have worse stability of microtubules in the basal ganglia.^11^

The majority of neurodegenerative disorders are proteinopathies, *i.e*., they are diseases of protein homeostasis with proteins misfolding and accumulating in aggregates, including PD.^13^ The high expression of neurofilaments in the non-active group when compared to the active one can suggest an increase in protein aggregation and PD progression, evidenced in our study with worsening of TH levels in non-active group. The mechanism by which neurofilaments aggregate is still unknown, but hyper-phosphorylation, which is the overexpression of neurofilaments, is considered one of the main triggers for neurofilaments (NF) aggregation,^13^ including higher levels of neurofilaments in the cerebrospinal fluid.^12^

A major limitation of this study is that there was no information on exercise intensity and duration per session, as well as information on how many years the participant walked for. This information could be relevant because in animal models, the type, duration, and frequency of exercise are associated with plastic responses in the nervous system.^35^ ^,^ ^36^ In our cross-sectional study, questionnaires were evaluated postmortem, which can cause information bias, though they were also validated for postmortem interview,^19^ and used before by other studies.^20^ ^,^ ^37^ ^,^ ^38^ Another limitation is the absence of control cases, future studies should include subjects without neurodegenerative disease neuropathology. Also a limitation is the presence of mixed neuropathology, since 75% of our sample had a significant number of neurofibrillary tangles. However, it is important to note that the Braak score for AD was similar between the non-active and active groups.

Despite the fact that our sample contains subjects with cognitive impairment defined as CDR≥0.5, there was no statistical difference between groups regarding the CDR. In a validation study of the postmortem interview, we found a high accuracy for normal cognition and moderate and advanced dementia; and lower accuracy was found for questionable and mild dementia.^19^ Although the information on cognitive impairment was used to describe the sample, it is important to highlight that the main study variable was LBP based on a neuropathological examination, which is not subject to bias related to postmortem evaluation. In addition, non-active and active groups were paired by age and gender, reducing the confounding chance by these variables.

In conclusion, in participants with similar LBP burden, classified as PD, an active lifestyle may improve dopamine synthesis and structural protein expression in the nigrostriatal system as well as decrease astrocyte activation, which are associated with neuronal death and the worsening of clinical symptoms in neurodegenerative diseases. Our study showed similar results to those found in animal models of PD that could explain the benefits of physical activity in the clinical improvement of PD patients who underwent exercise protocols. Future studies in human brain samples that evaluate the frequency, duration, and intensity of exercise are important to better understand the clinical effects of exercise on PD pathophysiology.
